# Characterization of β-Lactamases and Multidrug Resistance Mechanisms in Enterobacterales from Hospital Effluents and Wastewater Treatment Plant

**DOI:** 10.3390/antibiotics11060776

**Published:** 2022-06-07

**Authors:** Christopher Mutuku, Szilvia Melegh, Krisztina Kovacs, Peter Urban, Eszter Virág, Reka Heninger, Robert Herczeg, Ágnes Sonnevend, Attila Gyenesei, Csaba Fekete, Zoltan Gazdag

**Affiliations:** 1 Department of General and Environmental Microbiology, Faculty of Sciences, University of Pécs, 7622 Pécs, Hungary; heninger.reka@gmail.com (R.H.); fekete@gamma.ttk.pte.hu (C.F.); 2 Department of Medical Microbiology and Immunology, Medical School, University of Pécs, 7622 Pécs, Hungary; melegh.szilvia@pte.hu (S.M.); kovacs.k.krisztina@pte.hu (K.K.); pal.agnes@pte.hu (Á.S.); 3 Bioinformatics Research Group, Szentágothai Research Centre, 7624 Pécs, Hungary; urban.peter@pte.hu (P.U.); herczeg.robert@pte.hu (R.H.); gyenesei.attila@pte.hu (A.G.); 4 Educomat Ltd., Iskola utca 12/A, 8360 Keszthely, Hungary; eszterandreavirag@gmail.com; 5 Department of Molecular Biotechnology and Microbiology, Institute of Biotechnology, Faculty of Science and Technology, University of Debrecen, Egyetem Square 1, 4032 Debrecen, Hungary

**Keywords:** hospital effluents, wastewater treatment plant, Enterobacterales, β-lactamases, multiresistance

## Abstract

Antimicrobials in wastewater promote the emergence of antibiotic resistance, facilitated by selective pressure and transfer of resistant genes. Enteric bacteria belonging to *Escherichia coli*, *Klebsiella pneumoniae*, *Klebsiella oxytoca*, *Enterobacter cloacae,* and *Citrobacter* species (*n* = 126) from hospital effluents and proximate wastewater treatment plant were assayed for susceptibility to four antimicrobial classes. The β-lactamase encoding genes harbored in plasmids were genotyped and the plasmids were sequenced. A multidrug resistance phenotype was found in 72% (*n* = 58) of *E. coli* isolates, 70% (*n* = 43) of *Klebsiella* species isolates, and 40% (*n* = 25) of *Enterobacter* and *Citrobacter* species. Moreover, 86% (*n* = 50) of *E. coli*, 77% (*n* = 33) of *Klebsiella* species, and 25% (*n* = 4) of *Citrobacter* species isolates phenotypically expressed extended spectrum β-lactamase. Regarding ESBL genes, *bla*_CTX-M-27_ and *bla*_TEM-1_ were found in *E. coli,* while *Klebsiella* species harbored *bla*_CTX-M-15_, *bla_CTX-M-30_*, or *bla*_SHV-12_. Genes coding for aminoglycoside modifying enzymes, adenylyltransferases (*aadA1, aadA5)*, phosphotransferases (*aph(6)-1d, aph(3″)-Ib)*, acetyltransferases (*aac(3)-IIa)*, (*aac(6)-Ib*), sulfonamide/trimethoprim resistant dihydropteroate synthase (*sul*), dihydrofolate reductase (*dfrA*), and quinolone resistance protein (*qnrB1*) were also identified. Monitoring wastewater from human sources for acquired resistance in clinically important bacteria may provide a cheaper alternative in regions facing challenges that limit clinical surveillance.

## 1. Introduction

Antimicrobial resistance presents a global challenge to the fight against infections in modern time [1]. Each year more than 670,000 infections are due to antibiotic resistant bacteria in the European Union/European Economic Area (EU/EEA) according to data from the European Antimicrobial Resistance Surveillance Network (EARS-Net), and approximately 33,000 people succumb to these infections [2]. It is projected that, close to 2.4 million people are likely to die globally in high-income countries by the year 2050 due to diseases caused by antibiotic resistant microorganisms [3]. The widespread use of antimicrobials in clinical practice to control infectious diseases, their application in veterinary medicine coupled with the discharge of non-treated pharmaceutical effluent into the environment results in selective pressure which is associated with the emergence and subsequent evolution of bacteria resistant to antibiotics [4]. Bacteria have shown the ability to develop antimicrobial resistance in response to stressors in the environment in the form of bioactive molecules, which include antibiotics, heavy metals, and disinfectants, among other biocides [5]. For instance, hospital effluent carries high bacterial loads and might contain sub-lethal concentrations of antimicrobial agents and their metabolites that enter wastewater and can facilitate the emergence and spread of resistance genes among bacteria [6]. In addition, wastewater treatment plants (WWTPs) contribute to the progression and persistence of antimicrobial resistant bacteria in the environment worldwide [7]. It has been demonstrated that wastewater treatment plants serve as sinks for high loads of antimicrobials, antibiotic resistant bacteria (ARB), and their genetic resistance determinants [8]. They also provide optimal conditions, including pH and temperature, which facilitate lateral gene transfer, capable of transforming commensal bacteria into reservoirs of resistance genes [9].

Members of the order Enterobacterales, which bear similar biochemical and genetic characteristics are ubiquitous and form a major part of gut microbiota [10]. Some of them, such as *Klebsiella pneumoniae, Escherichia coli, Proteus, Citrobacter,* and *Enterobacter* cause infections including in the urinary tract, bloodstream, and respiratory tract (hospital and health-care associated pneumonia), as well as intestinal and intra-abdominal infections [11,12]. Enterobacterales exhibit a wide range of resistance attributed to either mutations in chromosomal genes or mobile genes captured from different source species by various mobile genetic elements and transferred to plasmids, which can shuttle between cells and confer or enhance resistance to certain chemical classes of antimicrobials that are frequently used against multi-drug resistant microorganisms [10]. Fluoroquinolones and β-lactam antibiotics, which include the sub-groups of penicillins, cephalosporins, and carbapenems are the most frequently prescribed antibiotics and preferred therapeutic choices against infections caused by members of Enterobacterales [13]. Hospitals and other environments characterized by high amounts of antibiotics are associated with multidrug resistant Gram-negative bacteria that have demonstrated an increasing resistance to those compounds [14]. Antimicrobial resistant genes (ARG), such as genes coding for extended-spectrum β-lactamases (ESBLs) and carbapenemases harbored by Enterobacterales and other Gram-negative bacteria are clinically significant and have been reported from hospital effluents and WWTPs [15,16]. These genes are typically encoded on plasmids which harbor mobile genetic elements such as transposons or integrons and genes known to encode resistance to other antimicrobial agents [17].

ESBLs are β-lactamases that are capable of hydrolyzing broad-spectrum cephalosporins and aztreonam, whose activity is inhibited by clavulanic acid [18]. More than 300 subtypes of ESBLs have been described and their evolution is believed to originate from common ancestral types: TEM-1, TEM-2, or SHV-1 [19]. Mutations occurring in those genes resulted in new β-lactamases which can hydrolyze extended-spectrum cephalosporins and aztreonam [20,21]. Enterobacterales are also known to express ESBLs different from TEM, or SHV related types, such as CTX-M-type β-lactamases which are encoded by genes captured on transferable plasmids and are among the most widespread ESBLs in Europe [22]. Carbapenems (imipenem, doripenem, ertapenem, and meropenem) are the most potent antimicrobials used to manage life-threatening infections caused by multiresistant Gram-negative bacilli and their efficacy has been diminishing since carbapenem-resistant Gram-negative strains have emerged following their extensive use [23]. Carbapenemase producers are resistant to almost all β-lactams and to other classes of antibiotics [24]. Their occurrence in the environmental matrices is increasing with hospital wastewater being reported as a key reservoir of carbapenemase-producing Enterobacterales [25]. Data on the prevalence of β-lactamase producing multiresistant Enterobacterales of clinical importance in wastewater from human sources in southwest Hungary is unavailable since most studies are centered on the clinical environment. This study was aimed at bridging this knowledge gap and was therefore designed to: 1. Evaluate the prevalence of β-lactam resistance in enteric bacteria found in urban wastewater covering hospital effluents, municipal wastewater, and WWTP; 2. Isolate enteric bacteria of clinical importance including *Klebsiella* species, *Escherichia coli*, and *Enterobacter* species and determine their antimicrobial resistance profiles; 3. Molecular typing and sequencing of plasmid DNA to establish the prevalence of β-lactamase enzymes from the isolates and to unearth other mechanisms conferring multiresistance. In this context, the findings contribute to important knowledge and are applicable in planning effective strategies to minimize the spread of multiresistance in the environment.

## 2. Results

### 2.1. Determination of Antibiotic Resistant Gram-Negative Bacteria

Bacteria grew in varying numbers on eosin methylene blue agar-ceftriaxone mixture (EMB-CRO) and eosin methylene blue agar-imipenem mixture (EMB-IMP) from all samples (Table 1). The average colony forming unit (cfu) count of bacteria growing on EMB-CRO from the hospital and the nursing home effluents was 1.6 × 10^5^, while the cfu count in the wastewater treatment plant and the municipal wastewater was 6.8 × 10^4^ cfu mL^−1^. Although the cfu count was 2-fold higher in the hospital effluents compared to the WWTP and municipal wastewater, no significant variation was observed in the cfu count on EMB-CRO among the sites (*p* = 0.532). The average cfu count of bacteria isolated on EMB-IMP from the hospital effluent and the nursing home was 4.3 × 10^4^, while the cfu count in the wastewater treatment plant and the municipal wastewater was 5.3 × 10^3^ cfu mL^−1^. These data show up to 8 orders of magnitude higher loads of bacteria capable of growing on EMB-IMP in hospital effluents and nursing home indicating a significant variation from that of the wastewater treatment plant and municipal wastewater (*p* = 0.003). The total cfu count of the bacteria was significantly higher (*p* = 0.0001) than the cfu count on both EMB-CRO and EMB-IMP.

The proportion of bacteria growing on EMB-CRO in relation to the total cfu count on EMB varied between 36% (H4) and 57% (H2) in the hospital effluents and the nursing home, and 49% (INFL) and 36.9% (DGSL) in the wastewater treatment plant, while it was 10.5% in the municipal wastewater. The proportion of bacteria growing on EMB-IMP in relation to the total cfu count on EMB was much lower than in the case of ceftriaxone. The EMB-IMP/EMB cfu ratios varied between 4.2% (NH) and 30.4% (H3) in the hospital effluents and the nursing home, compared to 3.5% (INFL) and 3.7% (DGSL) at the wastewater treatment plant. The lowest prevalence was found in municipal wastewater (0.1%). Whereas the EMB-CRO/EMB and EMB-IMP/EMB cfu ratios fluctuated in all the hospital effluent samples from the different sources, the resistance was observed to increase as the treatment progressed from the activated sludge reactor to the digested sludge for both antibiotics.

### 2.2. Characterization of Antimicrobial Resistant Enterobacterales

A total of 126 isolates were recovered from the samples and identified with MALDI-TOF MS. The isolates belonged to *E. coli*, 46% (*n* = 58), *Klebsiella pneumoniae*, 20.6% (*n* = 26), *Klebsiella oxytoca*, 13.5% (*n* = 17), *Enterobacter cloacae*, 7.1% (*n* = 9), *Citrobacter freundii*, 11.11% (*n* = 14), *Citrobacter braakii,* 0.8% (*n* = 1), and *Citrobacter amalonaticus,* 0.8% (*n* = 1). The isolates were obtained from the following samples: 63.49% (80 strains) from the hospital effluents, 8.7% (11 strains) from nursing home, 20.6% (26 strains) from wastewater treatment plant, and 7.1% (9 strains) from municipal wastewater. Other isolates identified as not belonging to the Enterobacterales (*Stenotrophomonas maltophilia n* = 19, *Elizabethkingia meningoseptica n* = 7, *Elizabethkingia miricola n* = 6, and *Acinetobacter junii n* = 1) were not of interest for this study and were excluded from the subsequent analysis.

### 2.3. Antimicrobial Susceptibility Profiles and Multiple Antibiotic Resistance Indices

The enteric bacteria demonstrated variable susceptibility to the tested antibiotics, with isolates from the hospital effluents and the nursing home showing a relatively higher resistance rate than isolates from the WWTP and the municipal wastewater (Table 2). The multiple antibiotic resistance index (MAR index) for an isolate was calculated as *a/b* where *a* is the number of antibiotics to which an isolate was resistant, and *b* is the total number of antibiotics against which the isolate was tested. The MAR index for a site was calculated as a/(b*c) where *a* is the aggregate antibiotics resistance score of all isolates from a sample, *b* is the total number of antibiotics tested and *c* is the number of isolates from sample. Isolates from H3 had the highest resistance rate (MAR index 0.683) among the hospital effluents. Those from the digested sludge were the most resistant (MAR index 0.560) among the wastewater treatment plant isolates, while municipal wastewater had the least resistant isolates (MAR index 0.444). *E. coli* demonstrated the highest MAR index (0.65) among the four genera, while *Citrobacter* spp. showed the lowest MAR index (0.39) (Table 3). A high prevalence of resistance (>80%) was observed for the third generation cephalosporins (3GCs) ceftriaxone (CRO), ceftazidime (CAZ), cefotaxime (CTX), and cefpodoxime (CPD), while significantly lower resistance rates were measured for carbapenems, imipenem, and meropenem (IMP and MEM) compared to the other antibiotics. From H1 and H2 samples, resistance to IMP was found in 20% and 8% of *Klebsiella* and *E. coli* isolates, respectively, and 1 (4%) *Klebsiella* isolate from H1 was resistant to MEM. Gentamicin (GEN) resistance was the least frequent among the three non-β-lactams.

The resistance rates between β-lactams (ceftriaxone, ceftazidime, cefotaxime, cefpodoxime, cefoxitin, imipenem, and meropenem) and the non-β-lactams (sulfamethoxazole/trimethoprim, gentamicin, and ciprofloxacin) antibiotics were not significantly different (*p* = 0.8550). However, a positive correlation was found between resistance in the two groups. Ceftriaxone resistance was positively correlated to SXT and CIP, ceftazidime resistance to SXT, cefotaxime to GEN, SXT and CIP, and cefpodoxime resistance to SXT and CIP. Notably, the *Enterobacter cloacae* and the *Citrobacter* spp. isolates were resistant to cefoxitin (a cephamycin-second generation cephalosporin), unlike the other genera.

**Figure 1:**
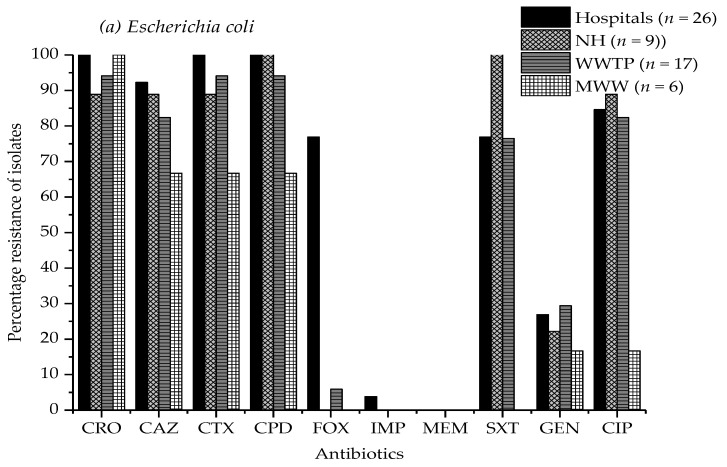

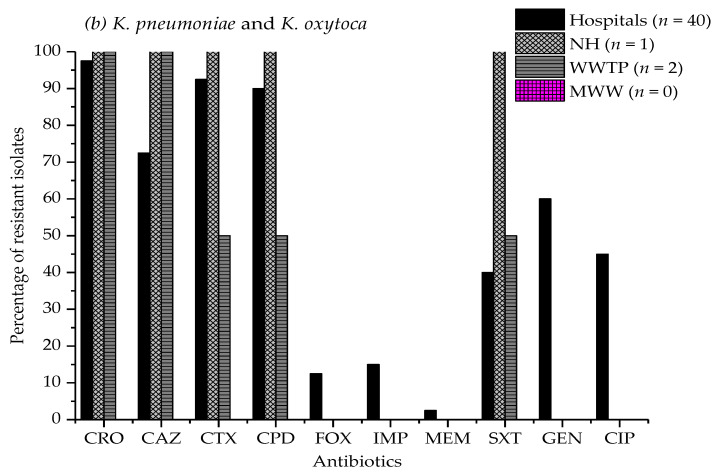

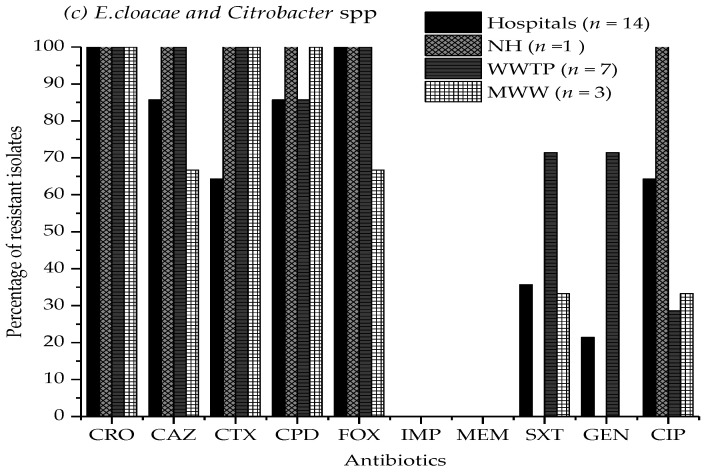
Antimicrobial resistance percentage of (**a**) *E. coli*, (**b**) *Klebsiella pneumoniae,* and *K. oxytoca*, (**c**) *Enterobacter cloacae*, and *Citrobacter* species isolates from four hospital effluents, nursing home, wastewater treatment plant, and municipal wastewater to ten different antibiotics. CRO, ceftriaxone; CAZ, ceftazidime; CTX, cefotaxime; CPD, cefpodoxime; FOX, cefoxitin; IPM, imipenem; MEM, meropenem; SXT, sulfamethoxazole/trimethoprim; GEN, gentamicin; CIP, ciprofloxacin. Study sites: NH, nursing home; WWTP, wastewater treatment plant; MWW, municipal wastewater. The absence of a bar indicates that no resistance was observed.

Figure 1a–c illustrate the antibiotic resistance patterns of *E. coli*, *K. pneumoniae,* and *K. oxytoca*, *E. cloacae,* and *Citrobacter* species isolates in the hospitals, nursing home, wastewater treatment plant, and municipal wastewater samples.

### 2.4. Multiple Antimicrobial Resistance and Co-Resistance

Multiresistance (defined as resistance to three or more classes of antibiotics) was observed in 65.08% (*n* = 82) of the isolates. 72.41% (*n* = 42) of *E. coli*, 69.77% (*n* = 43) of *Klebsiella* species, and 40% (*n* = 10) of *Enterobacter* and *Citrobacter* species isolates showed multiple drug resistance (MDR) phenotype, respectively. Although most of the strains were resistant to at least two antibiotic classes, resistance to three and to four chemical classes of antibiotics was observed (Table 4). Resistance to four chemical classes was only observed in 11 (19%) *E. coli* isolates, 3 (7%) *K. pneumoniae* isolates, and 2 (13%) *C. freundii* isolates. Four of the isolates resistant to four chemical classes showed resistance to 8 of the 10 antibiotics. The highest rate of multiple drug resistance (≥3) was reported for hospital effluents and nursing home, while the least was observed in municipal wastewater. The activated sludge reactor had the highest rate of MDR isolates in the wastewater treatment plant.

The most common associated/co-resistance was found among β-lactams, fluoroquinolone (CIP) and sulfonamide (SXT), while associated resistance to fluoroquinolone (CIP), aminoglycoside (GEN), and sulfonamide (SXT) was less common (Table 5). Although co-resistance was common among three chemical antibiotics classes, it also occurred for four chemical classes. Notably, associated resistance against 3GCs, CIP, and SXT was more frequent among *E. coli* and *Klebsiella* isolates. The highest rate of resistance to cephalosporins and fluoroquinolone (CIP) classes was reported in the four hospital samples, while that of cephalosporins and sulfonamide (SXT) occurred in the nursing home effluent samples.

### 2.5. Phenotypic Expression of β-Lactamases

Combined disc test of two different antibiotics and their β-lactamase inhibitor combinations were used to classify the isolates as extended-spectrum β-lactamase (ESBL) positive. Cefotaxime/clavulanic (CTC 40) and cefpodoxime/clavulanic acid (CD 01) markers defined 87 isolates (69.05%) as ESBL producers (Table 6). 62.07% (*n* = 54), 25.3% (*n* = 22), and 6.9% (*n* = 6) of ESBL-positive isolates originated from hospital effluents, wastewater treatment plant, and municipal wastewater, respectively. All *Enterobacter cloacae* isolates were confirmed to be non-ESBL-producing and showed 100% resistance to cefoxitin, which is associated with AmpC cephalosporinase activity. The isolates resistant to imipenem and/or meropenem were confirmed to be metallo-β-lactamase negative by phenotypic test.

### 2.6. Molecular Characterization of ESBL and Carbapenemase Genes

The ESBL-positive isolates were confirmed to harbor *bla*_CTX-M_ (100%) and *bla*_TEM_ 72.4% (*n* = 63) with PCR. The *NheI* digestion of the *bla*_SHV_ PCR product indicating the GLy238 → Ser mutation was positive in 17.2% (*n* = 15) of the samples (Table 7). Additionally, 69% (60 out of 87) of the isolates harbored both *bla*_CTX-M_ and *bla_T_*_EM_. This co-occurrence of *bla*_CTX-M_ and *bla*_TEM_ was observed in *E. coli* (62%), *Klebsiella* spp. (49%), and *Citrobacter* spp. (19%). In 17.2% of *Klebsiella* spp. Isolates, both *bla*_CTX-M_ and *bla_S_*_HV_ genes occurred simultaneously. Furthermore, 11.5% of the total isolates harbored the three groups of β-lactamase genes (*bla*_CTX-M_, *bla_TEM_*, and *bla*_SHV_). The broad-spectrum β-lactamase producers were more widespread in hospital and the nursing home effluents (68.9%) than in wastewater from other sources (WWTP, 24.1%, and municipal wastewater, 6.9%). None of the carbapenemase genes *bla*_VIM_, *bla*_IMP_, *bla*_KPC_, *bla*_OXA-48_, and *bla*_NDM_ was detected in the plasmid DNA of the carbapenem resistant isolates (*n* = 2, *E. coli*, and *n* = 7, *Klebsiella* species). However, carbepenem resistant *K. oxytoca* isolates were shown to carry the *bla*_VIM_ gene in the genome by a robust colony PCR test (Figure S1).

### 2.7. Next Generation Sequencing of Plasmids

**Figure 2:**
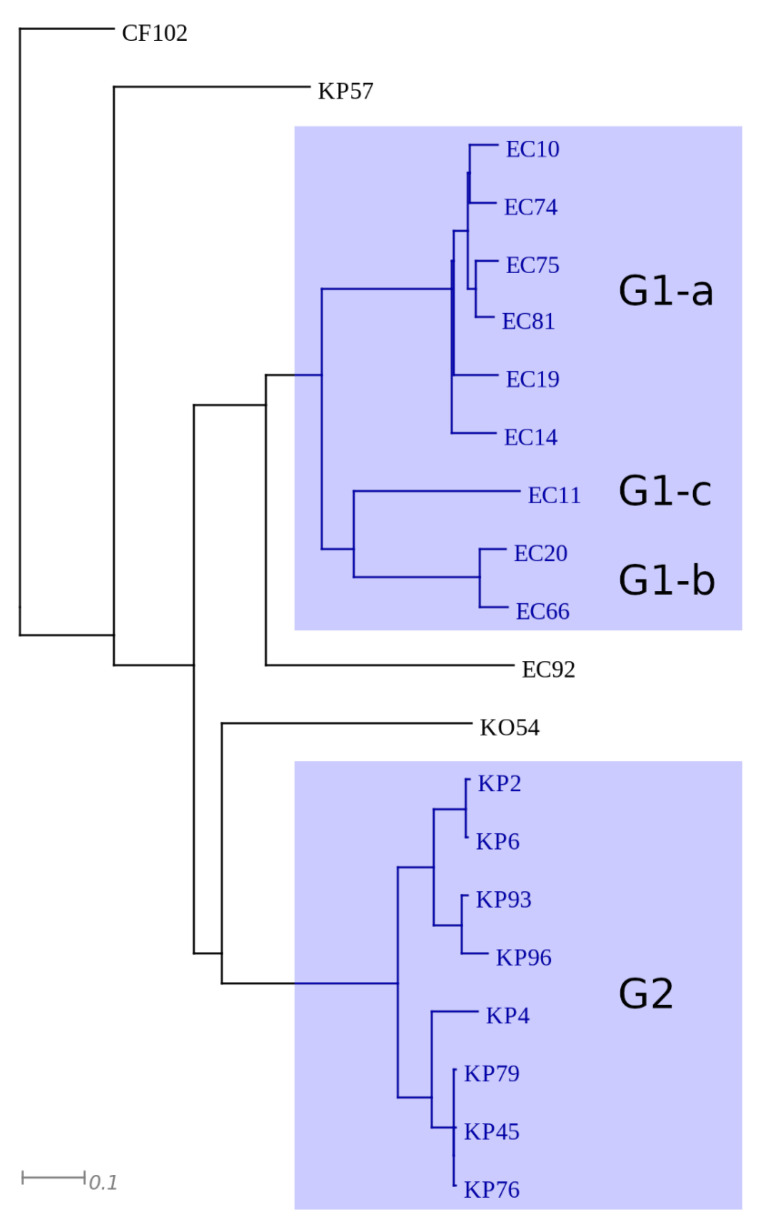
Representation of clusters based on MUMi distance of plasmid sequences. The major clusters are highlighted with blue.

Selected isolates belonging to *E. coli* (*n* = 10), *Klebsiella* spp. (*n* = 10)*,* and *Citrobacter* spp. (*n* = 1) were subjected to next generation sequencing and de novo assembly of plasmid sequences. Number, total length, and N50 of assembled contigs ranged between 17 and 64, 182 477–547 810 bp, and 11 403–62 343 bp, respectively (Table S1). According to maximal unique and exact match (MUM) indices the isolates were clustered into six groups, designated as G1–G6 (Figure 2). The two main groups contained the majority of *E. coli* (G1) and *K. pneumoniae* (G2) isolates and the four minor groups (G3–G6) enclosed single, unrelated isolates. Cluster G1 could be subdivided into 3 subgroups (G1-a, G1-b, and G1-c).

All isolates (*n* = 9) of G1 were identified as *E. coli* and were shown to harbor *bla*_CTX-M-27_ type ESBL gene, aminogylcoside (*aadA5, aph(3″)-Ib, aph(6)-Id*), folate inhibitor (*dfA17, sul1, sul2*), tetracycline (*tet(A)*), macrolide (*mph(A)*), and quaternary ammonium compound (*qacE∆*) resistance genes (Table S1). The *dfrA17*, *aadA5, qacE∆,* and *sul1* genes were part of a class I integron. The integron and the *mph(A)* gene were co-located on identical contigs in each isolate (Table S2). Besides, *aph(6)-Id, aph(3″)-Ib,* and *sul2* were also found to be co-localized in all isolates, but the contigs carrying them differed according to the subgroups (Figure S2 and Table S2). In subgroup G1-a (Figure S2a,b), a *Tn*2 transposon carrying *bla*_TEM-1_ was inserted between *aph(6)-Id* and *aph(3″)-Ib* and either *floR* or *tet(A)* was located upstream from this genetic structure. In subgroup G1b-G1c (Figure S2c and Table S2) *tet(A)* was located upstream from *aph(6)-Id, aph(3″)-Ib,* and *sul2*. The presence of *floR* and *tet(A)* was shown for subgroup G-1a and all subgroups of G1, respectively. Different plasmid incompatibility groups where characteristic for the three subgroups: in subgroup G1-a IncB/O/K/Z, IncFIA, IncFIB, and IncFII, in subgroup G1-b IncFIA, IncFII, and IncI, and in subgroup G1-c IncFIA, IncFIB, IncFII, and IncI was detected (Table S1).

Cluster G2 enclosed eight, closely related *K. pneumoniae* isolates. The presence of multiple β-lactamase genes (ESBL: *bla*_CTX-M-27_, non-ESBL: *bla*_TEM-1_, *bla*_OXA-1_), aminoglycoside (*aac(6′)-Ib-cr*, *aph(3″)-Ib,* and *aph(6)-Id*), chloramphenicol (*catB3*), folate inhibitor (*dfrA14, sul2*), and quinolone (*qnrB1, aac(6′)-Ib-cr*) resistance genes was characteristic for all isolates of G2. The *dfrA14* was carried by a class I integron which lacked the 3′ conserved sequence. The contigs carrying the aforementioned resistance genes where highly identical in all isolates (Table S2). Additionally, three isolates also harbored *aac(3)-IIa* on identical contigs. Two plasmid incompatibility groups (IncFIB and IncFII) were identified in this cluster (Table S1).

The four unrelated isolates, namely *C. freundii* CF102, *K. pneumoniae* KP57, *K. oxytoca* KO54, and *E. coli* EC92, were shown to harbor either *bla*_CTX-M-15_, *bla*_SHV-12_, *bla*_CTX-M-30_, or *bla*_CTX-M-1_ ESBL genes, respectively. Besides, a diversity in plasmid incompatibility groups and multiple antibiotic resistance genes (Table S1) were identified in these isolates, except for EC92 in which only the ESBL gene was detected. Genes *aac(6′)-Ib*, *aadA1*, *bla*_OXA-9_, and *bla*_TEM-1_ were located on a transposon (*Tn*1331) in KP57 and KO54 (Figure S3). In addition to *Tn*1331, KP57 also harbored *aph(3″)-Ib* and *aph(6)-Id* aminoglycoside resistance genes. In CF102 *aac(3)-IIa*, *bla*_OXA-1_, *catB3*, and *aac(3)-IIa* were identified on contigs that were highly similar to those found in *K. pneumoniae* isolates of G2 (Table S2). Moreover, CF102 also carried *aph(3″)-Ib*, *cmlB1*, and *tet(A)* genes as well.

## 3. Discussion

Hungary ranks among the countries with the lowest antimicrobial drug consumption rate (defined daily dose per 1000 inhabitants per day) both in hospitals and in the community sector in the European Union/European Economic Area based on the annual European Surveillance of Antimicrobial Consumption Network (ESAC-Net) report [26]. Despite the low consumption, disposal of untreated hospital effluents containing antimicrobials or their metabolites may select for the development of antibiotic resistant bacteria based on our findings. This study indicated a remarkable concentration of bacteria resistant to extended-spectrum cephalosporins in hospital effluents, nursing home and the WWTP, with bacterial cfu count on EMB-CRO being 2-fold higher in hospital effluents and nursing home than in the WWTP and the municipal wastewater. These data suggest either selection of resistance or the likelihood of bacteria of fecal origin carrying resistance traits from the source population being present in hospital effluents discharged into the wastewater network, following antimicrobial usage in the facilities. In a related study, cephalosporin resistant bacteria were also more concentrated in hospital wastewater compared to WWTPs [27]. Other studies have found an increase in antibiotic resistant bacteria in hospital wastewater networks, which has been attributed to large-scale antimicrobial usage in the hospital setting and the presence of their residues, especially at sub-inhibitory concentrations over extended periods [28,29]. A significant increase in the cfu count of imipenem resistant bacteria was also observed in hospital effluents relative to the WWTP. However, this was attributed to the presence of other Gram-negative bacteria, most notably non-fermenting *Stenotrophomonas maltophila,* which was frequently detected in the hospital samples and possesses an intrinsic resistance to imipenem.

We observed a more or less similar rate of resistance across the hospital and the nursing home effluents, measured by the multiple antibiotic resistance indices (MAR index), despite a huge variation in the bed capacity. H2 and H3, with the lowest bed capacities (106 beds and 127 beds, respectively), recorded high MAR indices (0.592 and 0.683). This may imply that the resistance rate largely depends on the regularly prescribed classes of antibiotics and the presence of different departments at each hospital as opposed to the number of patients accommodated in the facilities. All the isolates and all the sites reported multiple antibiotic resistance index values higher than 0.2. MAR index values greater than 0.2 indicate a high level of antibiotic contamination at the source [30]. The elevated MAR index values observed in *E. coli, Klebsiella* species, *E. cloacae,* and *Citrobacter* species are consistent with the MAR index values reported in the same members of Enterobacterales isolated from urinary tract infections in a tertiary-care hospital in Hungary in a surveillance study conducted between 2008 and 2017, where *E. coli* and *Klebsiella* species reported higher MAR index values compared to CES (*Citrobacter*, *Enterobacter,* and *Serratia*) [31]. Notably, there was an enrichment of the ARB in the sewage sludge after thermophilic digestion (MAR index 0.560, from 0.5000 in the activated sludge). The detection of increased antibiotic resistant bacteria in wastewater treatment plants’ effluent has been reported in other studies, [7,14,32]. However, the increase in resistance development among susceptible bacteria facilitated by WWTP processes has not yet been established [33].

Available data suggest that β-lactam agents (especially penicillins and cephalosporins) are the most frequently used class of antibacterial agents across Europe in both hospital and community settings [34,35]. Although our study used ceftriaxone to screen for the β-lactam resistant bacteria, high resistance rate to other third-generation cephalosporins (cefpodoxime, cefotaxime, and ceftazidime) was attributed to cross-resistance. High levels of resistance to the same antimicrobial agents in Enterobacterales were found in effluents from WWTPs in Navarra, Northern Spain [36]. The *bla*_CTX-M_ type extended-spectrum β-lactamase (ESBL) observed in *E. coli*, *K. pneumoniae,* and *C. freundii* in this study was largely responsible for resistance to extended-spectrum cephalosporins as reported in previous studies [22]. When comparing the plasmid sequences from 21 selected isolates, a cluster of *bla*_CTX-M-27_ harboring *E. coli* and another group of *bla*_CTX-M-15_ carrying *K. pneumoniae* isolates were revealed. In Hungary, CTX-M-15 and CTX-M-27 are found to be the dominant ESBL types among clinical isolates of *K. pneumoniae* and *E. coli,* respectively, which is in correspondence with our findings [37,38]. Considering that the *bla*_CTX-M-27_ harboring *E. coli* and *bla*_CTX-M-15_ carrying *K. pneumoniae* isolates identified in the hospital and nursing home effluents can be of fecal origin from patients and nursing home residents, it can be presumed that their dominance in our samples resembles their prevalence among local inhabitants. The highly identical contigs shared within a cluster raises the possibility of clonal relatedness of the isolates. Unfortunately, this question could not be addressed, because the DNA samples subjected to next generation sequencing was enriched for plasmids, and therefore the coverage of chromosomal fragments was too low to be suitable for MLST analysis. Besides the two major clusters, CTX-M-15 producing *C. freundii*, CTX-M-1 producing *E. coli*, SHV-12 producing *K. pneumoniae,* and CTX-M-30 producing *K. oxytoca* were also detected in our study. The majority of the isolates carried multiple antibiotic resistance genes, and many of these genes occurred to be co-located on defined contigs. These findings might explain the high frequency of associated/co-resistance and elevated MAR indices revealed in this study.

The ESBL producers were observed more frequently in hospital effluents and WWTP, which appears to mirror similar observations made in South Africa, Tunisia, and Spain, reporting high rates of ESBL prevalence from hospital effluent and urban wastewater treatment plants [39,40]. The presence of a high proportion of ESBL producers observed among isolates from hospital effluents may suggest increased prescription of certain extended-spectrum ß-lactam antimicrobials. Hsu et al. observed a significant increase in prescription of certain extended-spectrum β-lactam antibiotics, which were associated with high levels of ESBL producers in hospital effluents in Singapore [41]. The *E. cloacae* species were non-ESBL producers and showed resistance to cefoxitin (a cephamycin), which can be supported by the observation that ESBL-producing *E. cloacae* are less prevalent and hence rarely reported as most *Enterobacter* species carry AmpC cephalosporinases, which are not inhibited by clavulanic acid [36].

Although carbapenemases were not reported in the plasmid DNA of our isolates, a *bla*_VIM_ gene was detected among the *Klebsiella oxytoca* isolates by colony PCR. This is in support of certain reports regarding the emergence of carbapenemase-producing *Klebiella* spp. from environmental samples [42,43,44]. *Klebsiella* species harboring the *bla*_VIM_ gene have been previously reported among isolates at the Clinical Centre University of Pécs [45], which is located within this same catchment area, suggesting that hospital effluents may be reservoirs of carbapenemase producers that can be linked to clinical sources. The high rate of susceptibility to meropenem observed in this study is consistent with a similar observation regarding low carbapenem resistance in Enterobacterales reported from wastewater treatment plants [36]. Clinical surveillance data in a tertiary care hospital in Hungary among Enterobacterales for the period 2004–2015 reported zero resistance to carbapenems; imipenem, meropenem, and ertapenem [46].

Fluoroquinolones hold the fifth position in the European antimicrobial market, with a maximum of 3.04 DDD (defined daily dose/1000 inhabitants) [34]. Our findings showed a high rate of resistance to ciprofloxacin, consistent with previously reported resistance to fluoroquinolones among isolates from various environmental compartments [47,48]. Increased resistance to fluoroquinolones among Enterobacterales from urinary tract infections has been reported in clinical surveillance data [31,46]. Consistent with our finding, a recent study in South Africa also found an increased rate of co-resistance between third-generation cephalosporins and fluoroquinolones in *Klebsiella* spp. [39]. Other studies have demonstrated remarkable co-resistance to fluoroquinolones and broad-spectrum cephalosporins among *E. coli* and *K. pneumoniae* isolated from wastewater [49]. The presence of the quinolone resistance protein *qnrB1* and the aminoglycoside modifying enzyme *aac(6’)-Ib-cr* variant associated with low-level fluoroquinolone resistance identified in *Klebsiella* isolates indicates that acquired genes contribute to fluoroquinolone resistance among *Klebsiella* spp. from the environment. Ciprofloxacin resistance among *E. coli* was, however, mainly attributed to accumulation of double serine mutations in the DNA gyrase and topoisomerase IV genes, as reported by Fuzi et al. [50], since they did not carry acquired resistance genes.

Similarly, resistance to gentamicin in this study occurred frequently among *Klebsiella* strains, which is consistent with a previous observation in *Klebsiella* spp. from wastewater treatment plants and hospital effluents in KwaZulu-Natal, South Africa [39]. *bla*_CTX-M_ harboring plasmids are often known to carry other genes of resistance, particularly to aminoglycosides, tetracycline, sulfonamides, and trimethoprim, suggesting co-selection, co-expression, and hence co-resistance [51]. This finding can be linked to plasmid encoded aminoglycoside modifying enzyme encoding genes, *aph(3’’)-Ib* and *aph(6)-1d* (phosphotransferases), *aadA1* and *aadA5* (adenylyltransferases), and *aac(3)-IIa* (acetyltransferase), which were identified in *E. coli* and *Klebsiella* spp, some of which are associated with gentamicin and tobramycin resistance. An increased rate of resistance to sulfamethoxazole/trimethoprim among the isolates can be associated with sulfonamide resistance genes (*sul1* and *sul2*) and *dfrA* (*dfrA14* and *dfrA17*) expressing dihydropteroate synthase and dihydrofolate reductases responsible for target replacement, conferring resistance to sulfonamides and diaminopyrimidines. A recent clinical surveillance data on *E. coli* from urinary tract infections indicated a high resistance rate of sulfamethoxazole/trimethoprim [31], implying that the resistance observed in wastewater isolates may be clinical in origin.

It is notable that even though the isolates originated from different spots of the wastewater system, they were found to carry more or less the same plasmids groups. The main plasmids were the IncF replicons and their subtypes (FIA, FIB, and FII) which were evident in all the sequenced isolates. Acquired antibiotic resistance genes in bacteria are frequently carried on plasmids, with F plasmids being the most common conjugal plasmids in Enterobacterales linked to antibiotic resistance (“R factors”) [52]. According to Stephens et al. antibiotic resistance genes have been found in plasmids with a narrow host range, including IncI complex replicons (Z, B/O, K, or I1), but the majority of antibiotic resistance genes were associated with F replicons, and in most cases, multiple subtypes of F replicons were found on the same plasmids [53]. F- and I-complex replicons are frequently found in association with conjugating plasmids [53]. ESBL genes, carbapenemase genes, genes coding aminoglycoside-modifying enzymes, and plasmid-mediated quinolone resistance (PMQR) genes are the most frequently described resistance genes on IncF plasmids [54].

Multiple studies have described multidrug resistance in Enterobacterales, which is in line with the high frequency of strains exhibiting multiple antibiotic resistance phenotype among our isolates. Resistance to three or four antimicrobial classes was observed, with the majority of the isolates recording resistance to three classes, while a few isolates mainly *E. coli,* showed resistance to the four antibiotic families tested. Our findings reflect those reported by Rabbani et al. where over 60% of *E. coli* isolates from untreated hospital wastewater were multidrug resistant [55]. Estrada-Garcia et al. 2005, reported multidrug resistance in approximately 58% of *E. coli* [56]. Similarly, in a study involving 40 strains of *E. coli* isolated from the liquid hospital waste at Chittagong Medical College Hospital in Bangladesh, all were found to be multi-drug resistant (≥4) [57]. Multiple antibiotic resistant *K. pneumoniae* from wastewater have also been reported in a recent study in KwaZulu-Natal, South Africa [39]. In the clinical environment similar to wastewater, surveillance data have reported a significant increase in MDR (≥4 antibiotic classes) among uropathogens, including *E. coli*, *K. pneumoniae,* and *P. mirabilis* [46]. Multiresistance has been associated with the co-occurrence of resistance genes on mobile genetic elements where traits for resistance to multiple antimicrobials occur in particular plasmids and the same mechanism happens to be active against a wide spectrum of antimicrobials [55].

Multiple antibiotic resistance phenotype occurred at high frequency in the hospitals and the nursing home where individuals are likely to be put on a treatment regimen on a regular basis. In a related study, the percentage of multiple drug resistance in *E. coli* was higher in a nursing home than in hospital effluents [58]. Co-resistance between cephalosporin and ciprofloxacin in this study was more frequent among isolates from the four hospitals, while that of cephalosporin and trimethoprim/sulfamethoxazole occurred more frequently among those from the nursing home. These findings may be related to antimicrobial drug prescriptions and demonstrate that antimicrobial drug resistant bacteria are likely to be selected in the human gastro-intestinal tract due to antimicrobial usage [59]. The discharge of untreated hospital effluent into the urban wastewater network for co-treatment with the rest of municipal wastewater at the WWTP before releasing it into the environment, which is a general practice across many countries in Europe [60], may be directly linked to the high resistance rate observed in the hospital effluents. Isolates of clinical origin may disseminate resistance to environmental microbes, although resistant isolates from hospital effluents have not been correlated with those of clinical origin [32]. Decay of residual antimicrobials in the environment over time, coupled with their increased dilution in the wastewater network, limiting the chances for selection, may be attributed to the relatively low resistance in the WWTP and municipal wastewater, respectively.

## 4. Materials and Methods

### 4.1. Study Sites and Sample Collection

**Figure 3:**
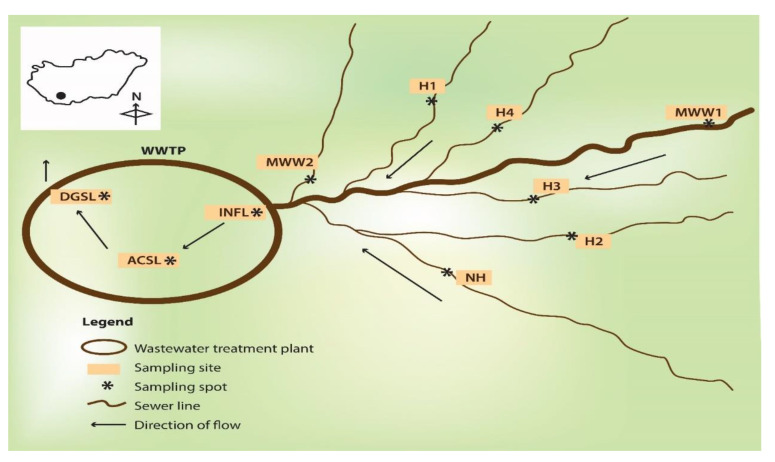
A schematic diagram depicting the sampling locations. H1–H4, hospital wastewater; NH, nursing home; MWW1 and MWW2, municipal wastewater; INFL, influent; ACSL, activated sludge; DGSL, digested sludge; WWTP, wastewater treatment plant.

This study was carried out in the city of Pecs, in southwest Hungary. Wastewater samples were drawn from four hospital wastewater discharge points, H1 (387 beds), H2 (106 beds), H3 (127 beds), and H4 (348 beds), a discharge point of a nursing home for the elderly (NH, 490 beds), municipal wastewater sewer lines (MWW), and a wastewater treatment plant (WWTP) (Figure 3). Effluent samples from the healthcare facilities were collected directly from two separate generation points serving different buildings before joining the main sewer pipe. A 30 mL sample was collected every 15 min by lowering a flask into the wastewater flow over a period of 4 h and the aliquots were pooled together to constitute a 480 mL composite sample in sterile 500 mL glass bottles. Samples from the WWTP were collected from the influent directly behind the grating screen. One grab sample was drawn from the activated sludge reactor and the digested sludge after thermophilic digestion. The municipal wastewater was collected 4 km upstream of the health care facilities (MWW1) and at a second spot upstream of the WWTP (MWW2), and was pooled. Samples were transported on ice to the laboratory and stored at 4 °C, before assaying within 6 h. The WWTP processes wastewater from the central business district, health care facilities, domestic wastewater, and some storm runoff and serves a population equivalent to slightly over 200,000 inhabitants. The wastewater treatment involves three stages (primary clarification, secondary-activated sludge system, and thermophilic sludge digestion, tertiary-UV treatment), with the final effluent discharged into the nearby surface stream. The study was conducted during 2019–2020.

### 4.2. Enumeration of Total and Antibiotic Resistant Gram-Negative Bacteria

Eosin methylene blue (EMB) agar (Biolabs, Budapest, Hungary) containing ceftriaxone (CRO, 2.0 μg mL^−1^; Merck, Darmstadt, Germany) or imipenem (IMP, 8.0 μg mL^−1^; Merck, Darmstadt, Germany) was used to enumerate the drug resistant enteric bacteria. The antibiotics were dissolved in appropriate diluents and filter sterilized through a 0.45 μm cellulose-acetate filter before addition to EMB. EMB agar plates containing no antimicrobials were used for determining the total bacteria count. A 5 mL subsample was drawn from a homogenized 480 mL sample and was serially diluted in phosphate buffered saline (PBS) containing 0.1% tween 80 up to 10^−3^ dilution. Aliquots of 50 μL were drawn from each dilution and plated on freshly prepared medium in triplicate. The plates were incubated under aerobic conditions at 35 ± 2 °C and colony formation was evaluated after 24 and 48 h [27]. Dilutions with 20–200 colony forming units (cfu) were enumerated and the number of bacteria was expressed as colony forming units per mL (cfu mL^−1^), while the resistance rate for each antibiotic corresponded to the ratio of cfu mL^−1^ on the culture medium with and without antibiotic [61].

### 4.3. Characterization of the Bacterial Isolates

On each sampling occasion, up to 5–10 lactose fermenting colonies of presumptive enteric bacteria representative of different colony morphotypes (colony contour, color, or size) were randomly picked. Typical green metallic sheen colonies were suggestive of *E. coli,* large mucoid pinkish colonies characteristic of *Klebsiella* species and pink to purple colonies typical of *Enterobacter* species [62]. The isolates were sub-cultured on nutrient agar, incubated at 35 ± 2 °C for further 18–24 h and were identified with MALDI-TOF MS. Mass spectrometry was performed using a Microflex MALDI Biotyper (Bruker Daltonics, Bremen, Germany) equipment. MALDI Biotyper RTC 3.1 software (Bruker Daltonics, Bremen, Germany), and the MALDI Biotyper Library 3.1 were employed for the spectrum analysis. Score values of ≥ 2.0 were considered reliable identifications [63]. For further characterization, the cultures were preserved at −80 °C in nutrient broth supplemented with 20% glycerol.

### 4.4. Antimicrobial Susceptibility Profiles and Phenotypic Detection of β-Lactamases

Antimicrobial susceptibility was established using the standardized disk diffusion method on Mueller Hinton agar (Biolabs, Budapest, Hungary) according to EUCAST 2018 guidelines. The standard antibiotic discs belonging to the following classes were used: (1) β-lactams; ceftriaxone (CRO, 30 µg), ceftazidime (CAZ, 10 µg), cefotaxime (CTX, 30 µg), cefpodoxime (CPD, 10 µg), cefoxitin (FOX, 30 µg), imipenem (IMP, 10 µg), and meropenem (MEM, 10 µg). (2) Aminoglycoside; gentamicin (GEN, 10 µg), (3) fluoroquinolone; ciprofloxacin (CIP, 5 µg), and (4) sulfonamide; sulfamethoxazole/trimethoprim (SXT, 1.25/23.75 µg) (Oxoid, Wesel, Germany). Quality control was performed using *E. coli* ATCC 25922, *Klebsiella pneumoniae* ATCC 180112, and *Enterobacter cloacae* ATCC 180083 as wild type negative controls while *E. coli* ATCC 151006, *Klebsiella pneumoniae* ATCC 180111, and *Enterobacter cloacae* ATCC 161002 were control strains with known resistance phenotype. Inoculum’s concentration was standardized to 0.5 McFarland turbidity, and the plates were incubated for 18–20 h at 35 °C and evaluated for the formation of inhibition zones. The zone diameters were interpreted based on the European Committee on Antimicrobial Susceptibility Testing clinical breakpoints, version 8.1, 2018 [64]. Multidrug resistance among the strains was defined as resistance to three or more antibiotic classes. A combined disk test was used to screen for the production of extended-spectrum β-lactamase. Cefotaxime (CTX 30 µg) and cefpodoxime (CPD 10 µg) (Oxoid, Wesel, Germany) disks were placed next to the disks with cefotaxime/clavulanic acid (CTC, 30/10 µg, CTC, 40) and cefpodoxime/clavulanic acid (10/10 µg, CD, 01). Similarly, carbapenem resistant isolates were screened for metallo-β-lactamase (MBL) production using a combined disk of imipenem/ethylene diamine tetraacetic acid (IMP 10 µg / EDTA 292 µg—IEL 292) (Oxoid, Wesel, Germany) on Mueller Hinton agar plates (Biolabs, Budapest, Hungary) with an inoculum of 0.5 McFarland. An increase in the inhibition zone size to ≥5 mm with the combined disks compared to the disk of cephalosporin/carbapenem alone was considered a positive test for β-lactamase production [65].

### 4.5. Molecular Typing of ESBL and Carbapenemases

Plasmid DNA was isolated by the alkaline lysis method using the Monarch plasmid DNA miniprep kit according to the manufacturer’s instructions (New England Biolabs T1010, Ipswich, Massachusetts, USA). DNA was isolated from freshly grown pure colonies transferred into Luria Bertani broth and incubated in an orbital shaker at 35 °C and 200 rpm for 12–16 h. All the centrifugation steps were carried out at 16,000× *g*. DNA concentration and purity were determined using a Nanodrop spectrophotometer (NanoDrop 2000, Thermo Scientific, Wilmington, NC, USA), and stored at –20 °C for subsequent PCR amplification. PCR reactions for selected genes belonging to *bla*_CTX-M_, *bla*_TEM_, *bla*_SHV_, *bla*_OXA_-_48_, *bla*_KPC_, *bla*_IMP_, *bla*_VIM,_ and *bla*_NDM_ families were performed in a final volume of 25 µL containing 12.5 µL DreamTaq PCR master mix (2×) composed of Dream Taq DNA polymerase, optimized 2× Dream Taq buffer, 4.0 mM, MgCL_2_, 0.4 mM each of dATP, dCTP, dGTP, and dTTP (Thermo Scientific, Waltham, MA, USA), 1.0 µM, Forward primer, 1.0 µM, Reverse primer, 1 µL template DNA, and by addition of nuclease free water. A conventional PCR assay was used and the amplification thermal profile was applied as follows: Initial denaturation at 95 °C for 2 min, 35 times repeated cycle of 95 °C for 30 s, 30 s at the appropriate primer annealing temperature for the specific primer, primer extension at 72 °C for 1 min, and final elongation at 72 °C for 10 min, with a holding step at 4 °C. Primers used and their corresponding annealing temperatures are as shown in Table 8. Gly238→Ser mutation associated with the hydrolysis of third-generation cephalosporins was identified through digestion of *bla*_SHV_ PCR product with *NheI* (New England Biolabs). Colony PCR was performed using OneTaq quick load Mastermix to determine the presence of chromosomally encoded metallo-β-lactamases among carbapenem resistant isolates. Individual colonies were dipped into the reaction tubes containing 25 µL One Taq master mix (New England Biolabs, Budapest, Hungary) PCR primers and nuclease free water. Thermal cycling conditions were initial denaturation at 94 °C for 2 min, 35 times repeated cycle of 94 °C for 30 s, 30 s at the appropriate primer annealing temperature for the specific primer, primer extension at 68 °C for 1 min, and final elongation at 68 °C for 10 min, with a holding step at 4 °C. Post PCR analysis was performed in 1.5% (*w*/*v*) agarose gel stained with 2 µL serva DNA stain G (Bio-Connect, Begonialaan, Netherlands). Then, 5 µL of each DNA sample was mixed with 2 µL of 6× loading dye and electrophoresed in 1× Tris—EDTA (TAE) buffer at 100 V for 1 h and visualized under an ultraviolet transilluminator. Next, 10 µL of either phage Lambda DNA digested with *EcoRI/HindIII* and a low range molecular weight marker (Thermo Scientific, Waltham, MA, USA) or both, in some cases, were included in each run as DNA size markers. In each PCR run, a positive control consisting of a clinical isolate of a confirmed reference strain was included for each genotype. The control strains were well characterized clinical isolates kindly provided by the microbiology laboratory of the University of Pecs Medical School [45]. For the robust colony PCR, 10 µL of each reaction was directly loaded onto an agarose gel alongside a PCR product from an appropriate reference strain and a DNA ladder.

### 4.6. Plasmid DNA Library Preparation and Sequencing

Selected isolates (*E. coli n* = 10, *K. pneumoniae n* = 9, *K. oxytoca n* = 1, and *C. freundii n* = 1) were subjected to NGS sequencing. The selection was based on antimicrobial susceptibility profiles and site of isolation. The library for NGS sequencing was prepared using Swift 2S Turbo DNA Library Kits (Swift Biosciences, Ann Arbor, Michigan, United States). Briefly, 100 ng genomic DNA was fragmented, end prepped, and adapter ligated. Magnetic bead size selection was performed to select 250–300 bp insert size fragments, followed by the library amplification according to the manufacturer’s instructions. The quality of the library was checked on the 4200 TapeSation System using D1000 Screen Tape (Agilent Technologies, Palo Alto, CA, USA) and the quantity was measured on Qubit 3.0. (Thermo Scientific, Waltham, MA, USA). Illumina sequencing was performed on the NovaSeq 6000 instrument (Illumina, San Diego, CA, USA) with a 2 × 151 run configuration. Quality control (QC), trimming, and filtering of 150 bp paired-end raw reads were performed in the preprocessing step. The QC analysis was performed with FastQC [70]. The Phred-like quality scores (Qscores) were set to >30. Poor quality reads, adapters at the ends of reads, limited skewing at the ends of reads were eliminated by using Timmomatic [71]. Since data contained genomic DNA debris, identification of plasmid-derived contigs was performed after de novo assembly of cleaned reads. For plasmid identification, genes characteristically encoded in plasmids for each strain were determined based on literature and by aligning them for the contigs using locally the Blast+ [72]. Prokaryotic gene finding was performed by Glimmer using the Bacterial, Archaeal, and Plant Plastid Code. Glimmer uses Interpolated Markov Models (IMMs) to identify the coding regions and to distinguish them from non-coding DNA, which enabled identified genes to be annotated [73]. Functional annotation and Gene Ontology (GO) analysis were carried out using OmixBox.Biobam as follows: sequences were blasted against the NCBI nr (non-redundant) database (taxID: 2Bacteria), applying blastn configuration locally. To retrieve GO terms associated with the 10 Hits obtained by the Blast search GO mapping and annotation were performed. GeneBank identifiers (gi), the primary blast Hit ids, were used to retrieve UniProt IDs making use of a mapping file from PIR (Non-redundant Reference Protein Database), including PSD, UniProt, Swiss-Prot, TrEMBL, RefSeq, GenPept, and PDB. Accessions were searched directly in the dbxref table of the GO database. BLAST result accessions were searched directly in the gene-product table of the GO database; GO annotations were specified according to GO terms: molecular function, cellular component and biological process [74]. For detection of antimicrobial resistance genes and identification of plasmid incompatibility groups ResFinder 4.1 and PlasmidFinder 2.1 were used [75,76]. Each contiq of all isolates was aligned with MUMmer 4.0 in order to identify similar regions [77]. MUM indices were calculated pairwise, and the resulting distance matrix was used to cluster the isolates with neighbor joining method [78]. Visualization of clusters was performed with Treesplits [79].

### 4.7. Statistical Analysis

A descriptive statistical analysis (mean, range, and percentage) was performed using Microsoft Excel 2013 (Redmond, WA, USA, Microsoft Corp.). OriginPro version 2016 (Northampton, Massachusetts, USA, OriginLab Corp.) was used for plotting and analysis. Shapiro–Wilk tests were performed to check the normality of variables, while one-way analysis of variance (ANOVA) was used to compare resistance rates among sampling locations. Pairwise t-test was performed to determine differences in resistance rates between hospitals and WWTP. A correlation matrix was used to examine the relationship between β and non-β-lactam antibiotic resistance. *p* values ≤ 0.05 were considered statistically significant.

## 5. Conclusions

Our findings demonstrate that wastewater from human sources may serve as an important reservoir of multiresistant Enterobacterales, including ESBL and carbapenemase producers, and it may be likely that some of these strains could be traced to clinical sources. Although β-lactam antibiotics are considered the backbone of antibiotic therapy, making them the most widely used antibiotics in clinical practice, they present a similar resistance rate among Enterobacterales from environmental sources as other classes of antimicrobials, namely fluoroquinolones, aminoglycosides, and sulfonamides, which can be linked to simultaneous transmission of plasmid encoded genes, creating a pool of multiresistant bacteria in the environment. Notably, multiresistant Enterobacterales harboring plasmid-mediated extended-spectrum β-lactamases primarily of CTX-M, TEM, and SHV types that degrade broad-spectrum cephalosporins are more common in hospital effluents and their presence can be attributed to the development of resistance in the source population, and/or its buildup in the aqueous environment through selection pressure as well as resistance dissemination of the phenotype via horizontal gene transfer. Besides the plasmid borne β-lactamases, metallo-β-lactamase VIM also contributes to the resistance phenotype. Additionally, the interpretative readings of the inhibition zones of cephamycin suggest the possible presence of endogenous AmpCs β-lactamases. The results also suggest that the population of multidrug resistant bacteria from the hospital and sanitary effluents that enters the WWTP is enriched during thermophilic digestion of the sewage sludge. The findings present a clear indication that acquired genes contribute to multiresistance in Enterobacterales from the wastewater environment, contrary to certain reports linking acquired resistance only to clinical isolates. Monitoring wastewater of anthropogenic origin is a promising strategy for generating valuable data that can be correlated to the prevalence of clinically important resistant bacteria from the source population and may provide a cheaper alternative in regions facing challenges that limit clinical surveillance.

## Figures and Tables

**Table 1:** The average concentration of bacteria growing on EMB, EMB-CRO, and EMB-IMP samples from hospitals, nursing home, wastewater treatment stages, and municipal wastewater samples.

Mean Colony Counts (CFU mL^−1^) *n* = 3					
Source	Total CFU Count EMB	EMB-CRO	CRO % Resistance	EMB-IMP	IMP % Resistance
H1	2.1 × 10^5^	9.95 × 10^4^	47.4	4.2 × 10^4^	20
H2	6.65 × 10^5^	3.8 × 10^5^	57	6.2 × 10^4^	9.3
H3	2.6 × 10^5^	1.42 × 10^5^	54.6	7.9 × 10^4^	30.4
H4	2.7 × 10^5^	9.8 × 10^4^	36	2.1 × 10^4^	7
NH	2.9 × 10^5^	1.17 × 10^5^	40.3	1.23 × 10^4^	4.2
INFL	1.01 × 10^5^	4.97 × 10^4^	49	3.6 × 10^3^	3.5
ACSL	7.1 × 10^5^	9.8 × 10^4^	13.8	4.6 × 10^3^	0.6
DGSL	2.3 × 10^5^	8.5 × 10^4^	36.9	8.6 × 10^3^	3.7
MWW	3.9 × 10^5^	4.1 × 10^4^	10.5	4.6 × 10^3^	0.1

EMB, eosin methylene blue agar without antibiotics; EMB-CRO, eosin methylene blue agar supplemented with ceftriaxone at 2.0 μg/mL; EMB-IPM, eosin methylene blue agar supplemented with imipenem at 8.0 μg/mL, cfu mL^−1^, colony forming units per milliliter. % resistance, ratio of cfu count on EMB-CRO and EMB-IMP expressed as a percentage of total bacterial cfu count on EMB from the hospital effluents (H1–H4), nursing home (NH), wastewater treatment plant (INFL-influent, ACSL-Activated sludge, DGSL-digested sludge), and the municipal wastewater (MWW).

**Table 2:** The antimicrobial susceptibility of enteric bacteria in percentage in the samples from the various sites.

Antimicrobial Susceptibility %												
Source	*n* = 126	CRO	CAZ	CTX	CPD	FOX	IMP	MEM	SXT	GEN	CIP	MAR Index
H1	27	100	81	100	100	19	20	4	57	44	78	0.596
H2	25	96	80	96	92	24	8	0	48	60	88	0.592
H3	6	100	100	100	100	50	0	0	100	33	100	0.683
H4	22	96	82	91	91	33	0	0	41	27	68	0.532
NH	11	100	100	92	82	18	0	0	91	27	82	0.591
INFL	9	100	100	78	67	22	0	0	56	22	56	0.500
ACSL	12	92	75	83	92	8	0	0	75	18	67	0.508
DGSL	5	100	100	100	100	40	0	0	40	40	40	0.560
MWW	9	100	78	100	100	33	0	0	11	11	22	0.444

Antimicrobial agents; CRO, ceftriaxone; CAZ, ceftazidime; CTX, cefotaxime; CPD, cefpodoxime; FOX, cefoxitin; IPM, imipenem; MEM, meropenem; SXT, sulfamethoxazole/trimethoprim; GEN, gentamicin; CIP, ciprofloxacin; *n*, number of isolates. MAR index, multiple antibiotic resistance index. Study sites: H1–H4, hospital effluent; NH, nursing home; INFL, influent; ACSL, activated sludge; DGSL, digested sludge; MWW, municipal wastewater.

**Table 3:** Antimicrobial susceptibility of each genus in percentage and their multiple antibiotic resistance indices (*n* = 126).

Isolates	*E. coli* (*n* = 58)		*Klebsiella* spp. (*n* = 43)		*E. cloacae* (*n* = 9)		*Citrobacter* spp. (*n* = 16)	
Antimicrobial Agent	R	S	R	S	R	S	R	S
CRO	58 (100)	0 (0)	42 (98)	1 (2)	9 (100)	0 (0)	14 (86)	2 (13)
CAZ	51 (88)	7 (13)	31 (72)	12 (28)	9 (100)	0 (0)	16 (100)	0 (0)
CTX	58 (100)	0 (0)	40 (93)	3 (7)	7 (78)	2 (22)	11 (69.)	5 (31)
CPD	58 (100)	0 (0)	38 (88)	5 (12)	8 (89)	1 (11)	14 (88)	2 (13)
FOX	2 (3)	56 (97)	5 (12)	38 (88)	9 (100)	0 (0)	15 (94)	1 (6)
IMP	2 (3)	56 (97)	6 (14)	37 (86)	0 (0)	9 (100)	0 (0)	16 (100)
MEM	0 (0)	58 (100)	1 (2)	42 (98)	0 (0)	9 (100)	0 (0)	16 (100)
SXT	43 (74)	15 (26)	16 (37)	27 (63)	5 (56)	4 (44)	2 (13)	14 (86)
GEN	15 (26)	43 (74)	24 (56)	19 (44)	0 (0)	9 (100)	5 (31)	11 (69)
CIP	46 79)	12 (21)	29 (67)	14 (33)	5 (56)	4 (44)	7 (44)	9 (56)
MAR index	0.646		0.551		0.555		0.394	

Antimicrobial agents; CRO, ceftriaxone; CAZ, ceftazidime; CTX, cefotaxime; CPD, cefpodoxime; FOX, cefoxitin; IPM, imipenem; MEM, meropenem; SXT, culfamethoxazole/trimethoprim; GEN, gentamicin: CIP, ciprofloxacin; R, resistant; S, susceptible; MAR index, multiple antibiotic resistance index.

**Table 4:** The number of chemical classes and antibiotics to which the isolates showed resistance among the 10 antibiotics.

Source	2 Classes	MDR (3 Classes)	4 Classes	7 + Antibiotics	10 Antibiotics
H1	93% (25/27)	78% (21/27)	0% (0/27)	15% (4/27)	0% (0/27)
H2	84% (21/25)	84% (21/25)	20% (5/25)	24% (6/25)	0% (0/25)
H3	100% (6/6)	83% (5/6)	33% (2/6)	50% (3/6)	0% (0/6)
H4	77% (17/22)	46% (10/22)	0% (0/22)	5% (1/22)	0% (0/22)
NH	92% (10/11)	73% (8/11)	27% (3/11)	27% (3/11)	0% (0/11)
INF	56% (5/9)	44% (4/9)	22% (2/9)	11% (1/9)	0% (0/9)
ACSL	75% (9/12)	67% (8/12)	17% (2/12)	17% (2/12)	0% (0/12)
DGSL	40% (2/5)	40% (2/5)	40% (2/5)	40% (2/5)	0% (0/5)
MWW	33% (3/9)	33% (3/9)	0% (0/9)	0% (0/9)	0% (0/9)

Study sites: H1–H4, hospital effluent; NH, nursing home; INFL, influent; ACSL, activated sludge; DGSL, digested sludge; MWW, municipal wastewater; MDR, multiple drug resistance. 2 classes refer to either a member(s) of the β-lactams (CRO, CAZ, CTX, CPD, FOX, IMP, and MEM) and one of the non-β-lactams, sulfamethoxazole/trimethoprim (SXT), aminoglycoside (GEN), and fluoroquinolone (CIP). 3 classes; either a member(s) of the β-lactams (CRO, CAZ, CTX, CPD, FOX, IMP, and MEM) and 2 antibiotics belonging to either sulfamethoxazole/trimethoprim (SXT), or aminoglycoside (GEN), or fluoroquinolone (CIP), or the 3 antibiotics belonging to the three non-β-lactam chemical groups (SXT, GEN), CIP). 4 classes; either a member(s) of the β-lactams (CRO, CAZ, CTX, CPD, FOX, IMP, and MEM) and all the other 3 classes, SXT, GEN, and CIP. 7+ antibiotics; more than 7 out of the 10 antibiotics tested. 10 antibiotics; the total number of antibiotics tested (CRO, CAZ, CTX, CPD, FOX, IMP, MEM SXT, GEN and CIP).

**Table 5:** The associated/co-resistance to the chemical classes of the antimicrobial agents (*n* = 126).

3 Chemical Classes	No.	%	4 Chemical Classes	No.	%
[β-lactam][CIP][SXT]	58	46	[β-lactam][CIP][GEN][SXT]	16	12.69
[β-lactam][CIP][GEN]	38	30.2			
[β-lactam][GEN][SXT]	19	15.1			
[CIP][GEN][SXT]	13	10.3			

CIP, ciprofloxacin (fluoroquinolone); SXT, sulfamethoxazole, (sulfonamide); GEN, gentamicin, (aminoglycoside).

**Table 6:** Prevalence of phenotypically expressed extended spectrum β-lactamases (ESBL) based on each genus.

Isolates	ESBL Negative (%) (*n* = 39)	ESBL Positive (%) (*n* = 87)	% ESBL Positive/Total Isolates (*n* = 126)
*E. coli* (*n* = 58)	8 (14)	50 (86)	40
*K. pneumoniae* (*n* = 26)	4 (15)	22 (85)	17
*K. oxytoca* (*n* = 17)	6 (35)	11 (65)	9
*E. cloacae* (*n* = 9)	9 (100)	0 (0)	0
*Citrobacter* spp. (*n* = 16)	12 (75)	4 (25)	3

**Table 7:** The number and percentage distribution and co-occurrence of *bla_CTX-M_*, *bla_TEM_*, and *bla_SHV_* genes among the ESBL positive isolates.

ESBL Gene Family	*E. coli*	*K. pneumoniae*	*K. oxytoca*	*Citrobacter*	Total (% of ESBL Positive, *n* = 87)
*bla*_CTX-M_	50 (86)	22 (85)	11 (65)	4 (25)	87 (100)
*bla*_TEM_	39 (67)	16 (62)	5 (29)	3 (19)	63 (72)
*bla*_SHV_	0 (0)	14 (54)	1 (6)	0 (0)	15 (17)
*bla*_CTX-M_ + *bla*_TEM_	36 (62)	16 (62)	5 (29)	3 (19)	60 (69)
*bla*_CTX-M_ + *bla*_SHV_	0 (0)	14 (54)	1 (6)	0 (0)	15 (17)
*bla*_CTX-M_ + *bla*_TEM_ + *bla*_SHV_	0 (0)	10 (38)	0 (0)	0 (0)	10 (11)
Total number (ESBL positive and ESBL negative)	58	26	17	16	126

**Table 8:** Sequences, annealing temperature, and expected product sizes of primer pairs targeting the specified β-lactamase genes.

Gene	Sequence (5′-3′)	Product Size (bp)	Annealing Temp (°C)	References
CTX-M-F CTX-M-R	TTTGCGATGTGCAGTACCAGTAA CGATATCGTTGGTGGTGCCATA	544	51	[66]
SHV-F SHV-R	ATGCGTTATATTCGCCTGTG GTTAGCGTTGCCAGTGCTCG	865	49	[67]
TEM-F TEM-R	GCGGAACCCCTATTTG ACCATTGCTTAATCAGTGAG	963	56	[68]
IMP-F IMP-R	GGAATAGAGTGGCTTAAYT TCGGTTTAAYAAAACAACCACC	232	52	[69]
KPC-F KPC-R	CGTCTAGTTCTGCTGTCTTG CTTGTCATCCTTGTTAGGCG	798	52	[69]
NDM-F NDM-R	GGTTTGGCGATCTGGTTTTC CGGAATGGCTCATCACGATC	621	52	[69]
OXA-48-F OXA-48-R	GCGTGGTTAAGGATGAACAC CATCAAGTTCAACCCAACCG	438	60	[69]
VIM-F VIM-R	GATGGTGTTTGGTCGCATA CGAATGCGCAGCACCAG	390	52	[69]

## Data Availability

The relevant data are provided along with the manuscript and additional information can be accessed at https://data.mendeley.com/datasets/j3mkwhzh84/1 Virág, Eszter; Mutuku, Christopher (accessed 4 May 2022), “Enterobacterales Plasmids”, Mendeley Data, V1, doi: 10.17632/j3mkwhzh84.1 “Plasmid sequences (1).rar” contains the identified plasmids, and “Omicsbox_annot_table.zip” contains the localization and annotation of the genes found in the given plasmids.

## Supplementary Materials

The following supporting information can be downloaded at: https://www.mdpi.com/article/10.3390/antibiotics11060776/s1, Table S1. Antimicrobial resistance genes and plasmid replicons detected by next generation sequencing. Table S2. Comparison of contigs carrying antimicrobial resistance genes. Table S3. EUCAST Clinical Breakpoint Tables v.8.1
